# *KIF5A* and *ALS2* Variants in a Family With Hereditary Spastic Paraplegia and Amyotrophic Lateral Sclerosis

**DOI:** 10.3389/fneur.2018.01078

**Published:** 2018-12-07

**Authors:** Marta Simone, Antonio Trabacca, Elena Panzeri, Luciana Losito, Andrea Citterio, Maria Teresa Bassi

**Affiliations:** ^1^Unit for Severe Disabilities in Developmental Age and Young Adults, Developmental Neurology and Neurorehabilitation, Scientific Institute IRCCS E. Medea, Brindisi, Italy; ^2^Laboratory of Molecular Biology, Scientific Institute IRCCS E. Medea, Lecco, Italy

**Keywords:** hereditary spastic paraplegias, amyotrophic lateral sclerosis, *KIF5A*, *ALS2*, Charcot-Marie-Tooth disease, infancy, adulthood

## Abstract

This paper describes the clinical evolution and the novel genetic findings in a *KIF5A* mutated family previously reported as affected by spastic paraparesis only. The additional evidence we report here, a homozygous *ALS2* mutation detected in the proband, and the clinical evolution observed in the affected members of the family, are in line with the evidence of an overlap between Hereditary Spastic Paraplegias and Amyotrophic Lateral Sclerosis associated with variants in these genes. The proband, a 14-years-old boy, started manifesting a pure form of HSP at age 14 months. The disease rapidly progressed to a juvenile form of ALS. This boy carries a heterozygous missense variant in *KIF5A* p.(Glu755Lys), inherited from the father, and a homozygous missense variant in the alsin protein encoded by the *ALS2* gene p.(Pro192Leu). The father shows a family history of ALS. In the last few years, he has been developing signs and symptoms of both upper and lower motor neuron degeneration, with mild bulbar motor involvement and emotional lability. The patients described in this family, confirm the continuum and partial overlap of the two clinical entities, HSP and ALS, historically viewed as distinct entities. The genetic findings in this family further substantiate the genetic bases underlying the overlap, broadening the clinical spectrum associated with *KIF5A* mutations.

## Introduction

Missense mutations within the kinesin family member 5A gene (*KIF5A*) are known causes of a dominant form of hereditary spastic paraparesis (spastic paraplegia type 10- SPG10, OMIM: 604187) and of Charcot-Marie-Tooth disease type 2 (CMT2) (1, 2). *KIF5A* encodes the neuronal kinesin heavy chain (KHC) implicated in the anterograde axonal transport (3). Recently, using a large-scale genome-wide association study and exome sequencing, *KIF5A* was described as a novel gene associated with Amiotrofic Lateral Sclerosis (ALS) (4, 5).

Mutations in *ALS2*, the gene coding for alsin protein, have been demonstrated (6) to be associated with a spectrum of rare autosomal recessive disorders including infantile ascending hereditary spastic paralysis (IAHPS), juvenile primary sclerosis (JPLS) with retrograde degeneration of the upper motor neurons, and juvenile ALS with both upper and lower motor neuron involvement. Alsin protein is a member of the guanine nucleotide exchange factors for the small GTPase RAB5 which affects endosome trafficking. Further evidence in one of the four different mouse models generated, indicate that the lack of alsin leads to selective defects both in mitochondria and Golgi apparatus, of the Corticospinal motor neurons (CSMN), thereby revealing the unique importance of alsin function for CSMN health and stability (7).

Here we report the clinical evolution and the novel genetic findings in a two generation family previously reported, with members affected by spastic paraparesis only and carrying a *KIF5A* mutation (1). The proband, a 14-years-old boy started manifesting an early onset (age 14 months) pure form of HSP rapidly progressing to a juvenile form of ALS. The boy has a family history for ALS, with the father manifesting, at present, only mild symptoms of ALS and a paternal uncle who died for ALS. The boy carries a homozygous variant in *ALS2* c.575C>T,p.(Pro192Leu) and a heterozygous variant in *KIF5A* c.2263G>A, p.(Glu755Lys); the latter is of paternal origin.

## Case Report

**Figure 1:**
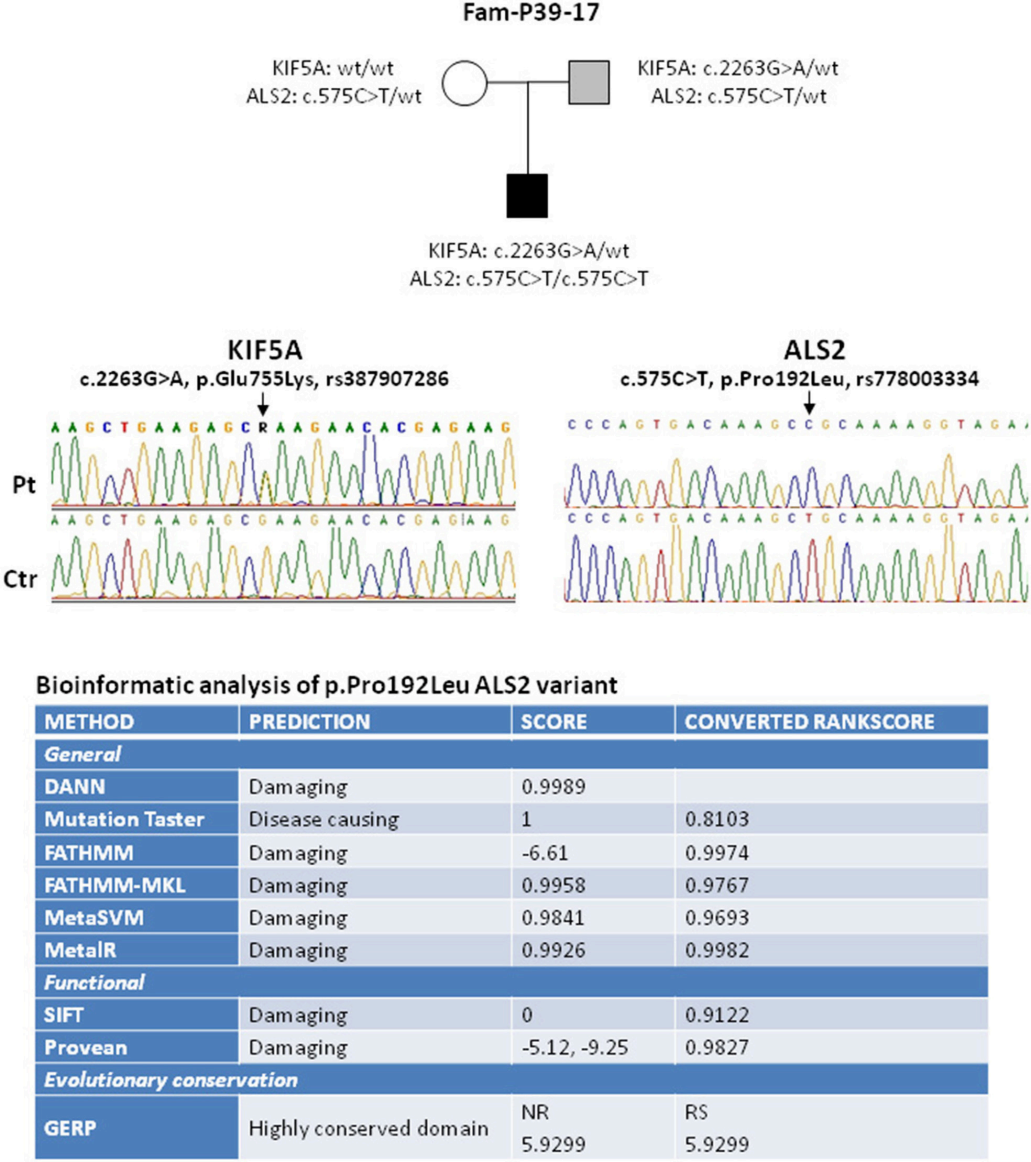
Elechtropherograms of the variants in *KIF5A* and *ALS2* identified in the proband and his family. In the upper panel the black box indicates the proband affected by the juvenile onset of ALS while the light gray box indicates the mild ALS clinical phenotype of the proband father. A summary of the bioinformatic analysis results of the ALS2 variant is reported in the bottom part. As listed in the figure, the software used provide prediction of negative effects at the general and functional level. GERP Software provide information about the evolutionary conservation of the substituted amino acid residue.

The proband of the family we report here, was born after an uneventful pregnancy to healthy, unrelated parents. At the 40th day of life, he underwent a cardiac surgery for severe aortic coarctation. A paternal uncle was affected by ALS (the diagnosis was supported by available clinical record reporting clinical and electrophysiological data) and he died at the age of 50 years before the patient came to our attention. The patient sat up unsupported at the age of 10 months and was able to walk at the age of 14 months, but he had abnormal gait with a progressive tendency to skidding and subsequent frequent falls. The child complained of easy fatigue and cramping at the lower limbs especially during the night. At the neurological examination, at age 4 years, he showed a paraparetic gait with lower limbs spastic hypertonia, enhanced deep tendon reflexes and bilateral ankle clonus and Babinski sign. Language skills developmental and cognitive functions were preserved. He underwent brain Magnetic Resonance Imaging, electromyography, somatosensory evoked potentials, blood tests, extensive search for metabolic disorders that resulted all negative, except for altered levels of muscle enzymes. At age 6 years, he underwent the first genetic screening for mutations in the most frequently mutated HSP genes available at that time (*SPG4, SPG7, KIF5A, REEP1, SPG11*). A missense mutation in the *KIF5A* gene was identified c.2263G>A, leading to an aminoacid change within the stalk domain, p.(Glu755Lys) (rs387907286, freq. 0.001219% in gnomAD Exomes). This variant was not found in 800 ethnically-matched controls from our internal database (48% females, 52% males) and this is in agreement with the low frequency of this variant in ExAC browser (8.256e-06, found only in European non-Finnish/Latino/South Asian females).The *KIF5A* variant is of paternal origin (Figure 1). At that time, the neurological examination of the father (aged 50 years) showed only subclinical sign of the disease with increased reflex at both upper and lower limbs. During the next few years, the disease slowly worsened in both the proband and his father.

The boy started developing weakness and spasticity in the upper limbs with distal fasciculations and painful muscle cramps, mild dysphagia and dysarthria. Electromyography showed signs of active denervation (fibrillation and positive sharp waves) and unstable fasciculation potentials. At present, the proband is 14 years old and reaches a score of 21 at the Spastic Paraplegia Rating Scale (SPRS), thus suggestive of moderate disability (8). In addition to the motor neuron signs, optical coherence tomography examination revealed the presence of a bilateral optic atrophy. Due to the progression of the disease and the symptomatic bulbar motor involvement, he underwent further genetic screening, by using a targeted next generation sequencing approach with a specific panel of 185 genes (including all known genes for HSP, ataxia, neuropathies and motor neuron disease including all known genes for familial ALS—the gene list is provided upon request). The screening detected a homozygous variant in the *ALS2* gene, c.575C>T, p.(Pro192Leu) (Figure 1). The variant frequency in gnomAD Exomes is 0.00164% (rs778003334). It has never been described in homozygous state and it was not found in 800 ethnically-matched controls from our database (48% females, 52% males), in agreement with its low frequency in ExAC browser (8.366e-06, found only in Latino/Other males and not present in the European population so far). Nine different prediction programs (DANN, Mutation taster, FATHMM, FATHMM-MKL, MetaSVM, MetaLR, SIFT, Provean, GERP) predict that the substitution of this evolutionary conserved residue in the N-terminus of the protein (RCC1-like domain) negatively affects protein function. Indeed, *in vitro* studies of another missense mutation in the RCC1-like domain, p.(Cys156Tyr), indicated that the aminoacid change reduced protein stability and led to loss of the protein (9). Further search for variant through a focused exome screening, performed on the proband and on both parents, failed to reveal additional nucleotide sequence changes with pathogenic significance. Both parents are obligate carriers of the *ALS2* variant and, as expected, the mother is healthy. The father carries both the *ALS2* and the *KIF5A* heterozygous variants; in the last 4–5 years, he started developing, initially focal, asymmetric signs and symptoms of affection of both the upper and lower motor neuron systems (fasciculations and muscular atrophy in upper limbs and spasticity and hyperreflexia in lower limbs), mild bulbar motor involvement (dysphagia) and emotional lability with no vegetative nor sensory symptoms. Electromyography showed signs of spontaneous electrical activity. He reached a total score of 36 at the ALS Functional Rating Scale-Revised (ALSFRS-R) which confirmed the initial progression of the disease (10).

## Materials and Methods

After obtaining an informed consent from the proband and the parents, genomic DNA was prepared from blood with standard method. The proband's DNA was screened by using a targeted next generation sequencing approach with a gene panel including 185 genes: all known causative genes for HSP, the known genes for recessive ataxia and for spinocerebellar ataxias, the most frequently mutated genes in neuropathies and the known genes for familial ALS (the gene list is available upon request). The targeted regions were designed to include coding exons with intronic 50 bp flanking sites and 3′ and 5′ untranslated regions (UTRs) by using the SureDesign system (Agilent Technologies, Santa Clara, CA, USA). The sequencing libraries were prepared from genomic DNA by using a Sure Select enrichment system (Agilent Technologies). Targeted libraries were run on MiSeq platform according to the manufacturer's instructions (Illumina, San Diego, CA, USA). ANNOVAR was used for annotation against the RefSeq database and the Single Nucleotide Polymorphism databases. The filtering strategy we applied led us to select only variants located in the coding regions, including the splice site, (synonymous variants were excluded), variants that exhibited a minor allele frequency of <1% or were not present in variant databases including those of the 1,000 Genomes Project, the Exome Aggregation Consortium (ExAC), the NHLBI exome sequencing project ESP6500. On average, 98.66 and 99.3% of bases were covered by at least 10 and 20 sequence reads, respectively. The mean read depth of the targeted regions was 824X. We used DANN, Mutation taster, FATHMM, FATHMM-MKL, MetaSVM, MetaLR to assess the general effects of the variants, SIFT and Provean for the functional effects and GERP for the evolutionary conservation of the variants. DANN is a pathogenicity scoring methodology based on deep neural networks (11). MUTATION TASTER (http://www.mutationtaster.org/) is an *in silico* prediction tool for the pathogenicity of a variant. FATHMM (Functional Analysis through Hidden Markov Models, http://fathmm.biocompute.org.uk/) is an *in silico* tool that predicts the effects of protein missense mutations based on a combination of sequence conservation and “pathogenicity weights”. FATHMM-MKL (http://fathmm.biocompute.org.uk/fathmmMKL.htm) predicts non-coding effects by integrating functional annotation information from the ENCODE. MetaSVM (12) is an ensemble score using Support Vector Machine (SVM) to integrate nine prediction scores and allele frequencies in 1KG database. MetaLR (13) is an ensemble score using Logistic Regression (LR) to integrate nine prediction scores and allele frequencies in 1KG database. SIFT (Sorts Intolerant From Tolerant, http://sift.bii.a-star.edu.sg/) is an *in silico* prediction tool for non-synonymous variants based on sequence homology derived from closely related sequences collected through PSI-BLAST. Provean (Protein Variation Effect Analyzer, http://provean.jcvi.org) is an *in silico* tool that predicts how non-synonymous or in-frame indel variant will affect a protein's biological function. GERP (Genomic Evolutionary Rate Profiling, http://mendel.stanford.edu/SidowLab/downloads/gerp/) is a conservation score calculated by quantifying substitution deficits across multiple alignments of orthologs using the genomes of 35 mammals. After filtering, we performed Sanger sequencing to confirm the variants detected through targeted sequencing analysis. The family trio (proband and both parents) was also rescreened for exome analysis by using the SureSelect Focused Exome (Agilent Technologies) with 6,100 known genes. The libraries of the trio were run on a NextSEQ500 (Illumina). Variant filtering and analysis were done as described above. A recessive model was first used and *de novo* variants were also searched.

## Discussion

In the family we describe here, the disease history of the boy started as almost pure HSP, affecting both lower and upper limbs, followed by a progressive involvement of the lower motor neuron. These features fit with the clinical picture shown by several patients with homozygous *ALS2* mutations. The onset as pure HSP, with involvement of the upper limbs, also correlates with the *KIF5A* variant we first detected, except for the early age of onset; this is indeed quite atypical for SPG10 patients. The bilateral optic atrophy progressively developed by the proband, is also included in the phenotypic spectrum of *KIF5A* complicated forms (14). Based on the recently demonstrated role of *KIF5A* in degeneration of both upper and lower motor neurons, it is hard to dissect the role of *ALS2* and *KIF5A* in the pathogenesis of the disease in the proband. Surely, the proband's father carrying both heterozygous variants in *KIF5A* and *ALS2*, and his family history for ALS might help in that. Indeed, the *ALS2* heterozygous variant being present also in the healthy mother, by itself, cannot be considered causative of the father's phenotype. Therefore, the *KIF5A* variant is likely the major contributor to the mild and slowly progressive form of ALS diagnosed in the father. Interestingly, it was demonstrated that *KIF5A* variants predominantly located in the N-terminal motor domain of *KIF5A* are causative for SPG10 and CMT2, whereas ALS-associated mutations are primarily located at the C-terminal cargo-binding tail domain (4). However, it is also possible that C-terminal and N-terminal variants act through a common mechanism that may lead to milder (i.e., SPG10) or more severe (i.e., ALS) phenotypes as previously suggested (4). The *KIF5A* variant p.(Glu755Lys) located in the stalk domain, was previously hypothesized having a destabilizing effect on protein dimerization since the Glu755 residue represents the *g* residue in the heptad repeats (α-helical coiled-coil repeats of seven amino acids each–*abcdefg-*_n_), of the stalk domain and mediates intra- and inter-helices ionic interactions with a stabilizing function (1). The destabilizing effect of the variant p.(Glu755Lys), at the functional level, can easily originate a sort of intermediate effect, in terms of diseases severity, as likely occurring in the father. In the proband, the intermediate effect of the *KIF5A* variant is instead likely masked by the potential stronger pathogenic effect of the *ALS2* variant, that can be therefore considered a likely mutation.

## Conclusion

The clinical and genetic findings described in this family, confirm the continuum and partial overlap of the two clinical entities, HSP and ALS, historically viewed as distinct ones. *KIF5A* represents part of the genetic bases underlying the overlap, based on large patient cohorts studies (4, 5) and single case report (an Asian family with HSP-*KIF5A* positive and pseudobulbar palsy, muscular fasciculations) (15). However, the genetic bases supporting the overlap of these motor neuron disorders are wider and include also another gene, SPG11-*SPATACSIN*. Mutations in *SPATACSIN* are indeed associated with the HSP-subtype, SPG11 and with the AR-juvenile ALS, termed ALS5 (16). More recently, association with an AR axonal Charcot–Marie–Tooth disease which shares some clinical characteristics with HSP and ALS was as also reported (17) suggesting that mutations in the *SPATACSIN* gene could cause a much wider spectrum of clinical features than previously recognized. In conclusion, this case shows an atypical genotype-phenotype correlation in HSP-*KIF5A*, broadening the clinical spectrum associated with HSP-*KIF5A* mutations. Considering the incomplete penetrance and the involvement of both upper and lower neurons, HSP-*KIF5A* should be considered in differential diagnosis and in the genetic work out of juvenile ALS. In addition, the co-occurence of variants in both genes *KIF5A* and *ALS2*, need to be checked as well, in these patients.

## Ethics Statement

Written informed consent to publish the report was obtained from each member of the family for the participation in the study and the publication of this report. The report describes the clinical evolution and the novel genetic findings of a family previously reported and approved by the Institutional Review Board and so it did not require a second approval.

## Author Contributions

MS and LL acquired the clinical data, reviewed the literature, and drafted the manuscript. AT and MTB designed the study, oversaw data acquisition, supervised the initial drafting, and critically revised the manuscript. EP and AC conducted the genetic analysis with MTB and contributed to manuscript writing analyzed the clinical data and critically revised the manuscript. All authors contributed to the interpretation of results and reviewed the final manuscript.

### Conflict of Interest Statement

The authors declare that the research was conducted in the absence of any commercial or financial relationships that could be construed as a potential conflict of interest.
